# An Up-to-date Approach to a Patient with a Suspected Autoinflammatory Disease

**DOI:** 10.5041/RMMJ.10277

**Published:** 2017-01-30

**Authors:** Merav Lidar, Eitan Giat

**Affiliations:** 1 Rheumatology Unit, Sheba Medical Center, Tel HaShomer Hospital, Tel Aviv, Israel; 2 Sackler School of Medicine, Tel Aviv University, Tel Aviv, Israel

**Keywords:** Autoinflammation, interleukin-1, familial Mediterranean fever, tumor necrosis factor-associated periodic fever syndrome, mevalonate kinase deficiency, cryopyrin-associated periodic fever syndrome, PFAPA

## Abstract

Autoinflammatory diseases (AID) are characterized by seemingly unprovoked self-limited attacks of fever and systemic inflammation potentially leading to amyloidosis. Familial Mediterranean fever (FMF) is the most common AID and therefore the most studied. Besides FMF, the other main hereditary AID are tumor necrosis factor-associated periodic fever syndrome (TRAPS), mevalonate kinase deficiency (MKD), and cryopyrin-associated periodic fever syndrome (CAPS). These hereditary diseases result from a mutant gene that is involved in the regulation of inflammation, resulting in a characteristic clinical phenotype. The differential diagnosis of AID can be challenging due to a wide overlap in clinical manifestations. Moreover, a considerable proportion of patients present with autoinflammatory symptoms but without a pathogenetic variant on genetic analysis. Furthermore, non-hereditary AID, such as the periodic fever, aphthous stomatitis, pharyngitis, adenitis (PFAPA) syndrome, which is the most common AID in children worldwide, must be excluded in certain circumstances. Herein we shall review the main AID and describe a practical approach to diagnosis in a patient with a clinical suspicion of AID.

## INTRODUCTION

The past two decades have brought a wealth of knowledge regarding the genetic background and the pathogenic mechanisms underlying many autoinflammatory diseases (AID). This newfound understanding assists us in making an earlier diagnosis and facilitates the correct diagnosis of atypical cases.

## FAMILIAL MEDITERRANEAN FEVER

The year 1997 was a seminal year for the AID. The gene for familial Mediterranean fever (FMF) was identified by two groups working independently and in parallel.^1^,^2^ It was allocated to the short arm of chromosome 16 and shown to code for an intracellular regulatory protein termed pyrin. Ever since this discovery, genetic testing has facilitated diagnosis in ambiguous cases and has widened the spectrum of clinical manifestations associated with disease.^3^ Moreover, the recognition of the PYRIN domain at the N terminus of pyrin in more than 20 human proteins involved in the regulation of inflammation ultimately led to the recognition of interleukin (IL)-1β as a central mediator of inflammation in FMF.^4^

Familial Mediterranean fever is characterized by recurrent episodes of fever and serositis. Patients typically develop high-grade fever, which lasts 1–3 days, associated with peritoneal inflammation, pleural involvement, attacks of arthritis, or erysipeloid erythema.^5^ Before the advent of genetic testing, typical cases were easily diagnosed based on the Tel HaShomer criteria^6^ (Table 1), while patients manifesting atypical disease were frequently dismissed. Today, patients presenting with “an inflammatory phenotype” (i.e. recurrent episodes of fever and additional manifestations of serositis, arthritis, or rash, which are suggestive yet not typical of FMF in character, localization, or duration) are referred to genetic testing. If two exon 10 mutations are identified (most commonly M694V, V726A, M694I, or M680I) a diagnosis of FMF is given with certainty. If only a single exon 10 mutation is found in an atypical case, additional corroboration is needed; this may take the form of a strong family history of FMF, the presence of exertional leg pain (calf or foot pain on seemingly mild exertion for an otherwise healthy person), or unequivocal response to a 3–6-month trial of oral colchicine. A single exon 10 mutation substantiated by minor clinical criteria, as described above, secures a diagnosis of FMF.^5^ Indeed, in addition to allowing for the diagnosis of atypical cases, the widespread use of genetic testing in populations with a high background frequency of FMF has taught us that the heterozygous state may be sufficient for the full clinical spectrum of FMF to be exposed. Specifically, in about one-third of our patients only a single MEFV gene exon 10 mutation is found.^5^

**Table 1:** Tel HaShomer Criteria for the Diagnosis of FMF.

Criteria	Description
Major Criteria	Peritonitis (generalized) Pleuritis (unilateral) or pericarditis Monoarthritis (hip, knee, or ankle) Isolated fever
Minor Criteria	Incomplete attacks affecting abdomen or lung or joints Exertional leg pain Response to colchicine

One major criterion or two minor criteria are required for a definite diagnosis of FMF.

Hence, in this age of genetic testing, our patient population includes atypical cases exhibiting, for example, only recurrent episodes of fever and exertional leg pain, patients manifesting recurrent pleuritis and pericarditis without abdominal attacks, and patients who have recurrent attacks with elevated acute-phase reactants but only rarely have an increase in body temperature.

Having secured a diagnosis of FMF, whether based on a typical clinical diagnosis befitting the Tel HaShomer criteria or consisting of a somewhat atypical presentation which in turn is corroborated by genetic testing, the question of treatment comes up. In the vast majority of cases colchicine suffices. A 1–3 mg/d dose in an adult eliminates or attenuates the frequency, duration, and intensity of attacks.^7^ About 10% of patients complain of persisting attacks despite colchicine treatment. Scrutinizing questioning unveils lack of compliance or intolerance in most cases of supposed inefficacy. Nevertheless, some 3%–5% of patients are indeed in need of other therapeutic options. What are the options available? Up until 2 years ago, our options consisted mainly of the addition of weekly intravenous colchicine to the maximal tolerated oral dose of the drug or a trial of tumor necrosis factor inhibitors in more severe cases.^8^ Both these options were based on small-scale open-label studies and case reports. They are still being used with variable success. However, delving into the role of pyrin in inflammation has unveiled the central role of the inflammasome in FMF as well as in other autoinflammatory diseases.^4^ The inflammasome functions as a platform on which pro-IL1β is cleaved into IL1β by caspase. Anti-IL1 agents, which block the effect of the excessive load of IL1 manufactured due to lack of caspase inhibition by the mutant pyrin, have surpassed all other therapeutic options for colchicine-resistant FMF patients.^9^ Two drugs are currently available in Israel, anakinra and canakinumab, while the third, rilonacept, may shortly be available as well. While differing in molecular structure and in mode of IL1β inhibition, as well as in the frequency of subcutaneous injection, these drugs show tremendous efficacy in eliminating attacks in colchicine non-responders. Lamentably, these drugs are not included in the Israeli “health basket” as yet but rather are issued on an individual basis in severe cases. The proposed criteria for approval of IL1 inhibitors in colchicine-resistant FMF are for patients with evidence of persistent proteinuria or biopsy-proven amyloidosis or in those suffering from more than one attack per month over a period of several months in association with objective signs of inflammation (such as an elevation of C-reactive protein levels during and between attacks). Importantly, as the efficacy of these agents in inhibiting amyloidosis has yet to be proven, it is mandatory to continue colchicine administration in unison with IL1 inhibition.

## TUMOR NECROSIS FACTOR-ASSOCIATED PERIODIC FEVER SYNDROME

Initially thought to represent “severe FMF” due to more prolonged episodes of inflammation (up to 3 weeks) and dominant inheritance pattern, familial Hibernian fever was renamed tumor necrosis factor-associated periodic fever syndrome (TRAPS) when culprit mutations were localized to the p55 tumor necrosis factor (TNF) receptor.^10^ In addition to fever, abdominal pain, pleurisy, and arthritis (symptoms which may be indistinguishable from those of FMF, apart from their duration), these patients suffer from characteristic periorbital edema and a migratory skin rash overlying fasciitis. Patients with TRAPS do not respond to therapy with colchicine. Mild cases may respond to non-steroidal anti-inflammatory agents, whereas more severe attacks may warrant medium-dose steroids. Unfortunately, as steroids do not prevent subsequent attacks, and as attacks may be quite prolonged, steroid toxicity is an issue. A score of case reports have described the efficacy of etanercept, a soluble TNF receptor, in the treatment of TRAPS. However, this drug is not effective in all patients, and its effect tends to wane over time.^11^–^13^ Importantly, use of anti-TNF monoclonal antibodies (e.g. infliximab) is not advisable due to the potential induction of a severe inflammatory attack.^14^ More recently, anti-IL1 therapy has proven useful in severe cases of TRAPS and has replaced etanercept as the preferred biologic treatment in this disease.^13^,^15^ Moreover, it has been suggested that those at higher risk of amyloidosis may benefit from earlier institution of IL1 inhibition.^16^

## MEVALONATE KINASE DEFICIENCY

Originally called hyperimmunoglobulinemia D syndrome (HIDS), mevalonate kinase deficiency (MKD) is a syndrome with attacks of intermediate duration (between the 3-day episodes of FMF to the prolonged 3-week episodes of TRAPS). Here, recessive mutations in the mevalonate kinase gene result in impaired mevalonate kinase activity and impaired isoprenoid biosynthesis that leads to autoinflammation, possibly through IL1 overexpression.^17^ The disease appears within the first decade of life, and attacks tend to be less frequent with age. Febrile episodes last between 3 and 7 days and are associated with cervical lymphadenopathy, abdominal pain, arthralgia, and a maculopapular skin rash.^17^ Non-steroidal anti-inflammatory drugs (NSAIDs) and corticosteroids may provide symptomatic relief during an attack. In more severe cases, IL1 inhibitor therapy is recommended for attack prevention. Colchicine and statins are not recommended due to inefficacy.^13^

## CRYOPYRIN-ASSOCIATED PERIODIC FEVER SYNDROME

The cryopyrinopathies encompass a spectrum of disease states including, from mild to severe, familial cold autoinflammatory syndrome (FCAS), Muckle–Wells syndrome (MWS), and neonatal onset multisystem inflammatory disease (NOMID) caused by autosomal dominant or *de novo* mutations in the NLRP3 gene. The clinical syndrome of FCAS includes cold-induced fever, urticaria-like rash, and constitutional symptoms. Muckle–Wells syndrome consists of fever, urticarial rash, and arthritis episodes, unrelated to cold exposure, lasting 1–7 days with the risk of progressive sensorineural hearing loss and amyloidosis developing over time, while NOMID runs a chronic course of fever, urticaria-like rash, epiphyseal overgrowth of long bones, and chronic aseptic meningitis with all its associated ailments. Interleukin-1-blocking agents are the mainstay of therapy of cryopyrin-associated periodic fever syndrome (CAPS).^13^ Studies in FCAS and MWS patients show a marked improvement in rash, headaches, fever, and joint pain as well as normalization of acute-phase reactants in 64%–97% of cases. Even in NOMID, IL1 blockade can reverse chronic inflammation including aseptic meningitis, papilledema, and cochlear inflammation, which otherwise ultimately culminates in hearing loss. As opposed to the other autoinflammatory syndromes herein discussed, there is no role for any biologic therapy other than IL1 inhibition in patients with CAPS.^13^

## PERIODIC FEVER, APHTHOUS STOMATITIS, PHARYNGITIS, ADENITIS

Although not a monogenic periodic fever, periodic fever, aphthous stomatitis, pharyngitis, adenitis (PFAPA) should be part of the differential diagnosis of a patient presenting with recurrent fevers. Here, patients suffer from attacks of fever, exudative pharyngitis, and aphthosis associated with enlarged cervical lymph nodes.^18^,^19^ Throat cultures are typically negative, and attacks recur despite preventive antibiotic therapy. Two typical features of the disease which point toward the correct diagnosis are the fixed periodicity (attacks occur every 3–6 weeks) and the prompt response to a single dose of steroids (2 mg/kg or a 20 mg dose of prednisone in an adult).^20^ Clinical manifestations, which appear in the majority of patients around the age of 4 years, tend to remit spontaneously after age 10 or at least decrease in frequency.^18^ Colchicine may reduce the frequency of the attacks.^21^,^22^ Whereas close to 1,000 children have been diagnosed with PFAPA at the Sheba Medical Center, fewer than 50 adults are being followed up in our outpatient clinic, on account of the high rate of spontaneous remission.

A summary of distinguishing clinical features and response to therapy of the common AID is detailed in Table 2.

**Table 2:** Distinguishing Clinical Features and Response to Therapy in AID.

	FMF	TRAPS	MKD	CAPS	PFAPA
**Mutant Gene**	MEFV	TNFRSF1A	MVK	NLRP3	Unknown
**Duration of Recurrent Fever**	1–3 days	3 weeks	4–7 days	FCAS, triggered by cold, usually 24 h; MWS, variable; NOMID, variable	Constant intervals (usually ~28 days); Median duration, 4 days
**Abdominal Pain**	Yes	Yes	Yes	Rarely	Sometimes
**Pleurisy**	Yes	Yes	No	No	No
**Arthritis/Arthralgia**	Yes	Yes	Yes	FCAS, no; MWS, NOMID, yes	Rare/absent
**Skin**	No	Yes	Yes	Yes	Rarely
**Ocular**	No	Yes	Yes	Yes	No
**Responds to Steroids**	No (with the exception of PFM)	Yes	Yes	Yes	Yes
**Response to Colchicine**	Yes	No	No	No	No/possible role in prevention
**Treatment**	Colchicine, anti-IL-1	Steroids, anti-TNF, anti-IL1	NSAIDs, steroids, anti-IL1	Anti-IL1	Steroids, tonsillectomy, anti-IL1

CAPS, cryopyrin-associated periodic fever syndrome; FCAS, familial cold autoinflammatory syndrome; FMF, familial Mediterranean fever; IL1, interleukin 1; MKD, mevalonate kinase deficiency; MWS, Muckle–Wells syndrome; NOMID, neonatal onset multisystem inflammatory disease; NSAIDs, non-steroidal anti-inflammatory drugs; PFAPA, periodic fever, aphthous stomatitis, pharyngitis, adenitis; PFM, protracted febrile myalgia; TNF, tumor necrosis factor; TRAPS, tumor necrosis factor-associated periodic fever syndrome.

## WHEN SHOULD THE PRESENCE OF HEREDITARY PERIODIC FEVER BE SUSPECTED?

Having delineated the most common monogenic hereditary periodic fevers, we should now attempt to establish in whom their presence should be suspected. The following five parameters should be utilized:

1. In the majority of cases, hereditary periodic fevers appear in early childhood and therefore pediatricians and family practitioners should be alert, especially when a positive family history or typical ethnic origin is present.
2. In the same pediatric population, rapid appearance of fever without signs or symptoms of a respiratory or a urinary tract infection.
3. Elevation of acute-phase reactants during attacks, with normalization of their levels during inter-critical intervals.
4. Complete well-being between attacks.
5. Repeated attacks and lack of seasonality: typically 4–6 attacks over an observation period of 9−12 months are needed when basing a diagnosis only on clinical manifestations—this period may be shortened by confirmatory genetic testing.

The diagnostic score available on the PRINTO website (http://www.printo.it/periodicfever/) is an evidence-based tool which aids general pediatricians in assessing patients with suspected periodic fevers. Patients with high diagnostic scores should be referred to specialized centers where genetic testing and therapeutic trials will be considered.

**Figure 1:**
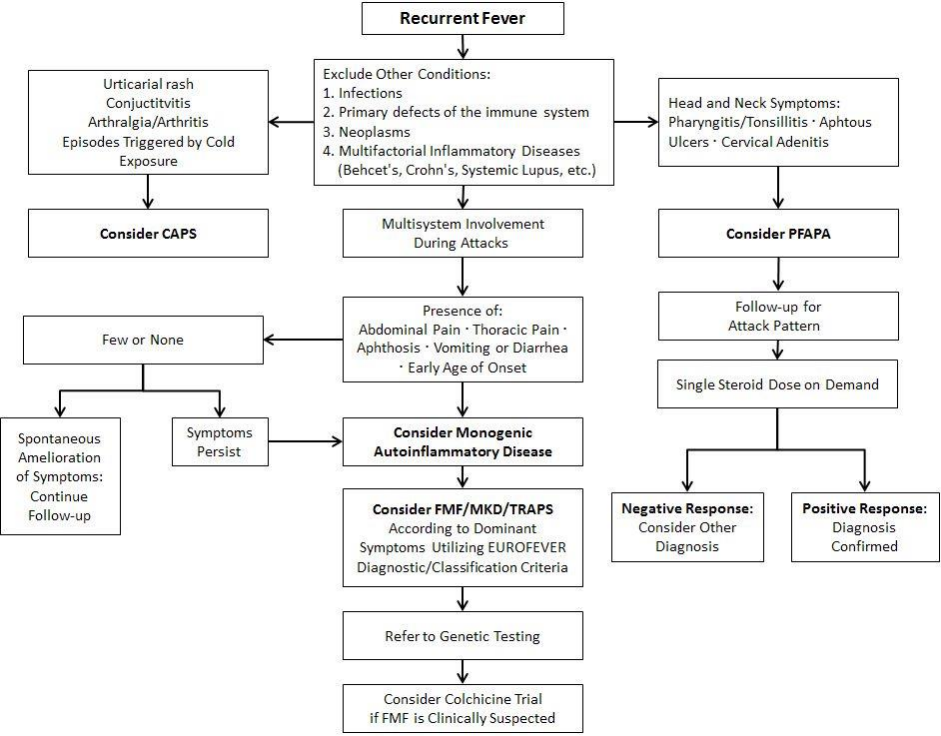
How to Diagnose an Autoinflammatory Disease (adapted from Federici and Gattorno 23).

An algorithm which serves to aid in the differential diagnosis of the common periodic fevers and PFAPA is presented in Figure 1.

In addition, the EUROFEVER diagnostic/classification criteria published by Federici et al. in 2015 highlight the prominent clinical manifestations of each and every disease.^24^ For example, according to these criteria episodes which last less than 2 days and attacks of chest or abdominal pain in a patient of Eastern or Northern Mediterranean origin are indicative of FMF, whereas the presence of aphthous stomatitis, an urticarial rash, enlarged cervical lymph nodes, or long episodes (>6 days) significantly reduce its probability. Periorbital edema, episodes with a duration of ≥6 days, the presence of a migratory rash, myalgias, and a strong family history (on account of the dominant inheritance pattern) increase the likelihood of TRAPS. An urticarial rash, sensorineural hearing loss, and conjunctivitis all point toward a diagnosis of CAPS, while exudative pharyngitis and the presence of abdominal pain during attacks are negative predictors of this disease.

The second decade of the twenty-first century has endowed us with a heightened awareness of autoinflammatory disease. This awareness coupled with improved tools for clinical and genetic diagnosis means that these disorders are now recognized earlier. Furthermore, the understanding of the paramount role of the inflammasome in these diseases and the development of biologic drugs that block its action have opened up new possibilities in the treatment of previously refractory patients.

## Abbreviations

AID: autoinflammatory diseases
CAPS: cryopyrin-associated periodic fever syndrome
FMF: familial Mediterranean fever
MKD: mevalonate kinase deficiency
MWS: Muckle–Wells syndrome
NOMID: neonatal onset multisystem inflammatory disease
PFAPA: periodic fever, aphthous stomatitis, pharyngitis, adenitis
TNF: tumor necrosis factor
TRAPS: tumor necrosis factor-associated periodic fever syndrome

## References

[1] French FMF Consortium (1997). A candidate gene for familial Mediterranean fever. Nat Genet.

[2] The International FMF Consortium (1997). Ancient missense mutations in a new member of the RoRet gene family are likely to cause familial Mediterranean fever. Cell.

[3] Sonmez HE, Batu ED, Ozen S (2016). Familial Mediterranean fever: current perspectives. J Inflamm Res.

[4] Chae JJ, Aksentijevich I, Kastner DL (2009). Advances in the understanding of familial Mediterranean fever and possibilities for targeted therapy. Br J Haematol.

[5] Lidar M, Livneh A (2007). Familial Mediterranean fever: clinical, molecular and management advancements. Neth J Med.

[6] Livneh A, Langevitz P, Zemer D (1997). Criteria for the diagnosis of familial Mediterranean fever. Arthritis Rheum.

[7] Ozen S, Demirkaya E, Erer B (2016). EULAR recommendations for the management of familial Mediterranean fever. Ann Rheum Dis.

[8] Koga T, Migita K, Kawakami A (2016). Biologic therapy in familial Mediterranean fever. Mod Rheumatol.

[9] van der Hilst JC, Moutschen M, Messiaen PE, Lauwerys BR, Vanderschueren S (2016). Efficacy of anti-IL-1 treatment in familial Mediterranean fever: a systematic review of the literature. Biologics.

[10] Aksentijevich I, Galon J, Soares M (2001). The tumor-necrosis-factor receptor-associated periodic syndrome: new mutations in TNFRSF1A, ancestral origins, genotype-phenotype studies, and evidence for further genetic heterogeneity of periodic fevers. Am J Hum Genet.

[11] Bulua AC, Mogul DB, Aksentijevich I (2012). Efficacy of etanercept in the tumor necrosis factor receptor-associated periodic syndrome: a prospective, open-label, dose-escalation study. Arthritis Rheum.

[12] Jacobelli S, André M, Alexandra JF, Dodé C, Papo T (2007). Failure of anti-TNF therapy in TNF receptor 1-associated periodic syndrome (TRAPS). Rheumatology (Oxford).

[13] Ter Haar N, Lachmann H, Özen S (2013). Treatment of autoinflammatory diseases: results from the Eurofever Registry and a literature review. Ann Rheum Dis.

[14] Nedjai B, Hitman GA, Quillinan N (2009). Proinflammatory action of the antiinflammatory drug infliximab in tumor necrosis factor receptor-associated periodic syndrome. Arthritis Rheum.

[15] Rigante D, Frediani B, Cantarini L (2016). A comprehensive overview of the hereditary periodic fever syndromes. Clin Rev Allergy Immunol.

[16] Cantarini L, Lucherini OM, Muscari I (2012). Tumour necrosis factor receptor-associated periodic syndrome (TRAPS): state of the art and future perspectives. Autoimmun Rev.

[17] Esposito S, Ascolese B, Senatore L (2014). Current advances in the understanding and treatment of mevalonate kinase deficiency. Int J Immunopathol Pharmacol.

[18] Thomas KT, Feder HM, Lawton AR, Edwards KM (1999). Periodic fever syndrome in children. J Pediatr.

[19] Hofer M, Pillet P, Cochard MM (2014). International periodic fever, aphthous stomatitis, pharyngitis, cervical adenitis syndrome cohort: description of distinct phenotypes in 301 patients. Rheumatology (Oxford).

[20] Vanoni F, Theodoropoulou K, Hofer M (2016). PFAPA syndrome: a review on treatment and outcome. Pediatr Rheumatol Online J.

[21] Tasher D, Stein M, Dalal I, Somekh E (2008). Colchicine prophylaxis for frequent periodic fever, aphthous stomatitis, pharyngitis and adenitis episodes. Acta Paediatr.

[22] Dusser P, Hentgen V, Neven B, Koné-Paut I (2016). Is colchicine an effective treatment in periodic fever, aphtous stomatitis, pharyngitis, cervical adenitis (PFAPA) syndrome?. Joint Bone Spine.

[23] Federici S, Gattorno M (2014). A practical approach to the diagnosis of autoinflammatory diseases in childhood. Best Pract Res Clin Rheumatol.

[24] Federici S, Sormani MP, Ozen S (2015). Evidence-based provisional clinical classification criteria for autoinflammatory periodic fevers. Ann Rheum Dis.

